# Changes in microbiome and metabolomic profiles of fecal samples stored with stabilizing solution at room temperature: a pilot study

**DOI:** 10.1038/s41598-020-58719-8

**Published:** 2020-02-04

**Authors:** Mi Young Lim, Seungpyo Hong, Bo-Min Kim, Yongju Ahn, Hyun-Jin Kim, Young-Do Nam

**Affiliations:** 1 Research Group of Healthcare, Korea Food Research Institute, Jeollabuk-do, 55365 Republic of Korea; 2 EZmass Co., Ltd., Gyeongsangnam-do, 52828 Republic of Korea; 3 Theragen Etex Bio Institute, Gyeonggi-do, 16229 Republic of Korea; 4 Department of Food Science and Technology, Division of Applied Life Sciences (BK21 Plus), Institute of Agriculture and Life Science, Gyeongsang National University, Gyeongsangnam-do, 52828 Republic of Korea; 5 Department of Food Biotechnology, Korea University of Science and Technology, Daejeon, 34113 Republic of Korea

**Keywords:** Metabolomics, Microbiome

## Abstract

The gut microbiome is related to various host health conditions through metabolites produced by microbiota. Investigating their relationships involves association analysis of the population-level microbiome and metabolome data, which requires the appropriate collection, handling, and storage of specimens. Simplification of the specimen handling processes will facilitate such investigations. As a pilot study for population-level studies, we collected the fecal samples from three volunteers and tested whether a single sample collection procedure, particularly using OMNIgene-GUT, can be used to reliably obtain both microbiome and metabolome data. We collected fecal samples from three young and healthy Korean adults, stored them at room temperature with and without OMNIgene-GUT solution up to three weeks, and analyzed their microbiome and metabolite profiles. We found that the microbiome profiles were stably maintained in OMNIgene-GUT solution for 21 days, and the abundance relationships among metabolites were well preserved, although their absolute abundances slightly varied over time. Our results show that a single sampling procedure suffices to obtain a fecal sample for collecting gut microbiome and gut metabolome data of an individual. We expect that the health effects of gut microbiome via fecal metabolites can be further understood by increasing the sampling size to the population level.

## Introduction

Changes in microbiome composition and metabolic function are associated with various diseases including obesity^1^, type 2 diabetes^2,3^, inflammatory bowel disease^4^, celiac disease^5^, and various cancers^6^. However, how the gut microbiome affects the host is not fully understood, although metabolites produced by gut microorganisms are presumed to mediate this interaction^7,8^. To investigate microbiome–metabolite–host relationships, it is necessary to collect both microbiome and metabolite information from the same individuals. Further, population-level data collection should be pursued to find such associations with strong statistical supports.

Although population-level microbiome analyses have been carried out in various countries^9–13^, there have been few studies collecting both microbiome and metabolome data, partly due to obstacles in fecal sample collection. Researchers need to recruit participants who are willing to collect their own fecal samples, pack the self-collected specimens, and deliver them to research facilities. To encourage participation, the sample collection methodology should be simple, and the collected sample ought to be easily sent to the research facility via conventional parcel delivery system. Meanwhile, changes to the microbial and metabolite profiles should be minimized.

For microbiome investigations, alteration of the microbiome profiles at room temperature^14^ is particularly problematic. This can be mitigated by removing sequences corresponding to bacteria that proliferate at room temperature^15^, a procedure adopted by the American Gut project^9^. Alternatively, fecal samples can be collected in stabilizing solutions, such as 95% ethanol, RNAlater, and OMNIgene-GUT, the utility in microbiome studies of which has been demonstrated in previous studies^14,16–18^. Fecal sampling methods for metabolome analysis have been studied separately from those for microbiome analysis. The storage methods currently recommended for metabolite analysis involve immediate aliquoting and freezing of the samples, and transportation of the samples at low temperature^19^, which are difficult to apply to population-based studies. If both microbiome and metabolite profiles can be simultaneously retrieved from a single fecal sample, the burden on participants can be reduced and researchers can recruit more participants. The possibility of obtaining both microbiome and metabolite information by a fecal collection method was recently reported^20^, yet it is unclear whether fecal metabolite profiles can be obtained from samples stored in a stabilizing solution at room temperature for a prolonged period, and whether it is appropriate to conduct integrated microbiome and metabolomics analysis from these samples.

In this pilot study, we investigated whether both microbiome and metabolite profiles can be retrieved from a single fecal sample collected in a stabilizing solution and stored at room temperature. We used the OMNIgene-GUT kit as the fecal collection method, because it is convenient for a user to collect a fixed quantity of fecal material with this kit and it includes stabilizing solution and a mixing ball in a tube, which allows immediate homogenization of a standard volume of fecal material and stabilizing solution at the point of collection. We examined microbiome and metabolome profiles from the three volunteers’ fecal samples that were stored in the OMNIgene-GUT solution at room temperature for up to three weeks, which we considered might be the time it takes to directly or indirectly deliver fecal samples to research facilities.

## Results

### Sample collection and experimental scheme

**Figure 1:**
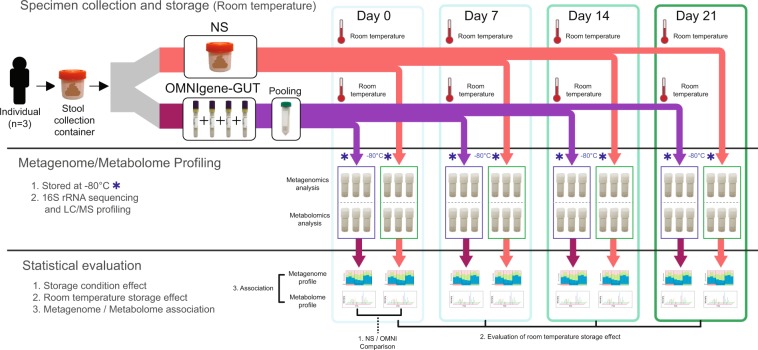
Sample collection and analysis. Fecal samples were collected from three young and healthy Korean adults. Each fecal sample was divided into two parts. For non-stabilized (NS) samples, the fecal sample was aliquoted into 24 cryotubes. For samples preserved with OMNIgene-GUT solution (OMNI samples), each fecal sample was mixed with OMNIgene-GUT solution using four OMNIgene-GUT tubes, pooled into one Falcon tube, and then aliquoted into 24 cryotubes. Samples were stored at room temperature until they were relocated to a deep freezer at four time points. The microbiome and metabolite profiles were analyzed for each specimen.

Fecal samples from three young and healthy Korean adults were collected for this study. The fecal sample from each participant was divided into two parts according to the preservation conditions: a non-stabilized (NS) sample and a sample preserved with OMNIgene-GUT solution (OMNI sample). In total, 144 aliquots (three participants × two preservation conditions × four storage durations × two different analyses × three replicates) were prepared. The aliquots were stored at room temperature, until they were relocated to a deep freezer (−80 °C) on day 0, 7, 14, or 21. After collecting all samples, we profiled the microbiome and metabolome of each sample. Subsequently, we investigated the differences in microbiome and metabolite profiles caused by storage condition and storage durations, and tested whether microbiome and metabolome associations could be detected reliably from the same specimens. The sample collection and experimental scheme are illustrated in Fig. 1.

### Microbiome community diversity

The utility of preservation solutions in microbiome studies has been reported in previous studies^14,17,18^, but we wanted to confirm the preservation ability of the OMNIgene-GUT solution before further analyses. To elucidate the effects of storage conditions on microbiome profiles, we conducted 16S rRNA gene sequencing on the 72 fecal samples.

**Figure 2:**
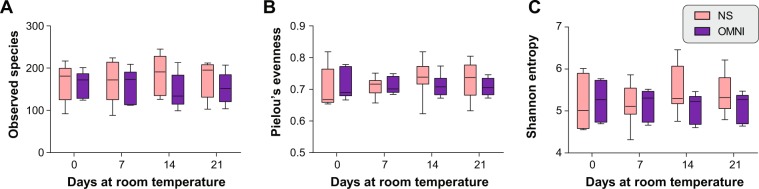
Comparison of microbial diversity between NS and OMNI samples at each time point. (**A**) Number of observed species. (**B**) Pielou’s evenness. (**C**) Shannon entropy.

First, we measured the microbiome diversity in each sample in terms of the number of observed species, Pielou’s evenness, and Shannon entropy. In the immediately (day 0) frozen samples, there was no statistically significant difference between samples preserved in the different conditions, OMNI and NS **(**Fig. 2**)**. In addition, no significant differences in diversity indexes between OMNI and NS groups were observed over the period of three weeks.

### Changes in microbial community structure

**Figure 3:**
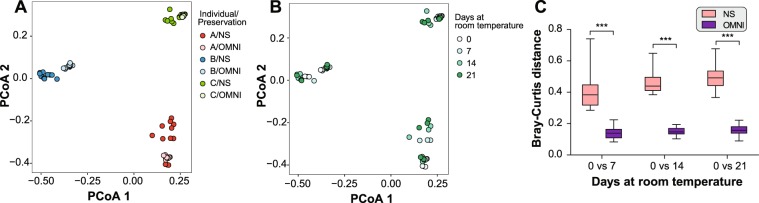
Microbial community structure based on Bray–Curtis distance. (**A**,**B**) Principal coordinates analysis (PCoA) was conducted, and samples were plotted on the first two principal coordinates, with colors for individual and storage condition (**A**) and for days stored at room temperature (**B**). (**C**) Bray–Curtis distance between immediately frozen and room temperature storage samples (***p < 0.001; *t*-test).

We then compared microbiota compositions of the samples using Bray–Curtis distances. In the principle coordinates analysis (PCoA) plot, samples from each individual were clearly separable from others regardless of storage condition and duration (Fig. 3A). Among samples from the same individual, OMNI samples were closely clustered, whereas NS samples tended to become scattered with prolonged storage (Fig. 3A,B). The high stability of microbiome composition in the OMNIgene-GUT solution was clearly observed as low Bray–Curtis distances between immediately frozen samples and those frozen at the other time points, while NS samples showed a high dissimilarity that increased over time (Fig. 3C). The same trend was observed in the profile of the top 20 most abundant genera **(**Fig. S1**)** and in the heat map of family-level abundance (Fig. S2).

**Figure 4:**
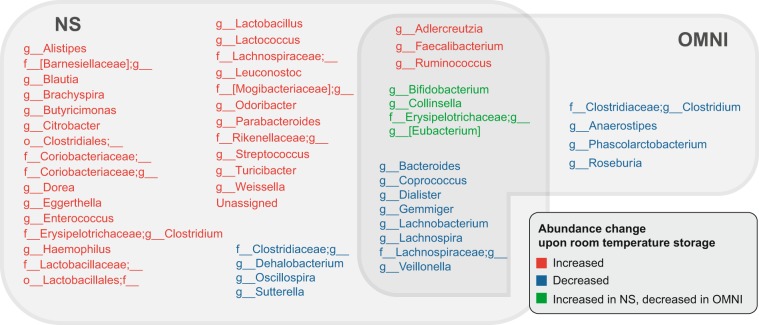
Changes in microbiome profiles at room temperature over time. For each storage condition, the taxa that significantly changed over time (FDR < 0.01; linear mixed model analysis) are presented. Red and blue colors indicate that the taxa increased and decreased over time, respectively. Green color indicates that the taxa increased in NS but decreased in OMNI samples.

To examine changes in microbial community structure over time in detail, we performed linear mixed model analyses for each storage condition separately. Among 104 genus-level taxa, the relative abundances of 35 genera increased and those of 12 genera decreased over time in NS samples after room temperature storage **(**Fig. 4**)**. In OMNI samples, the relative abundance of three genera increased, while those of 16 genera decreased **(**Fig. 4**)**. Under both storage conditions, the relative abundances of *Adlercreutzia*, *Faecalibacterium*, and *Ruminococcus* increased and those of eight taxa including *Bacteroides*, *Dialister*, *Lachnospira*, *Lachnobacterium*, and *Veillonella* decreased. The relative abundance of taxa including *Bifidobacterium* and *Collinsella* was changed in both NS and OMNI samples, but in opposite directions. We further inspected changes in microbial abundance by calculating log_2_-fold changes in the relative abundance at each time point compared to that in the immediately frozen samples. The number of genera with absolute log_2_-fold changes >1 was greater in the NS samples than in the OMNI samples **(**Fig. S3**)**. These results indicate that application of the OMNIgene-GUT solution in sample storage at room temperature reduces changes in microbial composition and can minimize bias caused by fecal sampling and storage in microbiome studies.

### Metabolites in immediately frozen NS and OMNI samples

**Figure 5:**
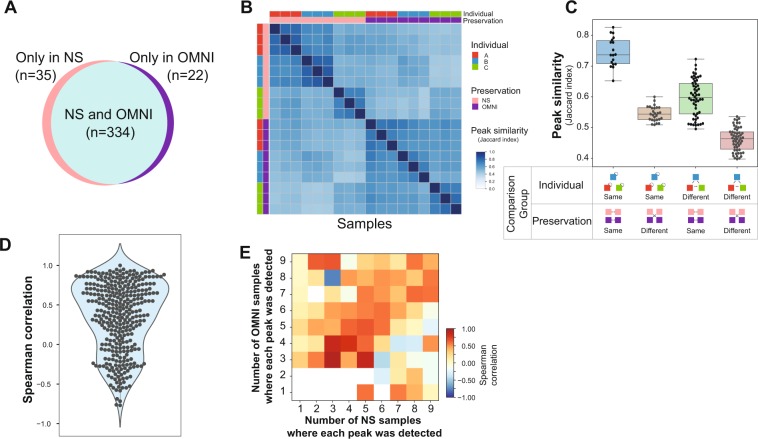
NS vs. OMNI peaks. (**A**) Number of metabolite peaks detected in the NS and OMNI samples. (**B**) Degrees of peaks commonly detected in the samples, measured by the Jaccard index. (**C**) Comparison of the Jaccard index according to individual and preservation condition. (**D**) Spearman correlation coefficients of peak intensities between the NS and OMNI samples. (**E**) Average Spearman correlation of peak intensities between NS and OMNI samples according to the numbers of NS and OMNI samples where a peak was detected.

We next investigated how preservation solution and room temperature storage affected metabolite profiles. The metabolites of 72 fecal samples were profiled using LC-MS analysis, and 391 unique metabolite peaks were detected. We first evaluated whether usage of the preservation solution changes the metabolite profiles by comparing the metabolite peaks detected in the immediately frozen NS and OMNI samples. When the intensity of a metabolite peak was larger than zero in a sample, the corresponding peak was considered to be ‘detected’ in the sample. We found that 334 (85%) metabolite peaks were detected in both NS and OMNI samples **(**Fig. 5A). This indicates that the kinds of metabolites detected from samples with and without preservative solution treatment are similar to each other. We then calculated the Jaccard index to measure the qualitative similarity of the detected peaks between each sample. The heat map of the Jaccard indices showed that the samples from each individual in the same preservation condition showed high similarity and that the samples in different preservation conditions showed low similarity **(**Fig. 5B**)**. For a clear demonstration, the similarities of detected peak sets were compared according to the preservation condition or the individuals **(**Fig. 5C**)**. The samples collected from the same individual and stored in the same preservation condition showed the highest level of similarity, sharing about 75% of the metabolite peaks with each other (Fig. 5C). The similarity of the samples that were collected from different individuals and stored in the same preservation solution was reduced. This result indicated that each individual possessed different types of fecal metabolites. A reduction in similarity between samples collected from the same individual but stored in different preservation conditions was observed. The differences in the metabolite peaks detected in NS and OMNI samples suggest that organic solvent in the OMNIgene-GUT solution may affect the elution of metabolites. We then evaluated the quantitative similarity of the metabolite profiles between NS and OMNI samples by calculating Spearman correlations of the intensities of each metabolite peak. About 44% of the metabolite peaks were shown to have a correlation coefficient of more than 0.5, and 34% showed statistically significant correlations at a false discovery rate (FDR) <0.2 (Fig. 5D). When a metabolite peak was detected in similar numbers of samples in NS and OMNI, the correlation coefficient of the metabolite peak between NS and OMNI samples was relatively high. Further, the peaks preferentially detected in the NS samples tended to show negative correlation with the corresponding peaks in the OMNI samples **(**Fig. 5E**)**. These results indicate that the OMNIgene-GUT solution may affect the type of metabolite peaks that can be detected, but the intensity relationships of the detected peaks in both the immediately frozen NS and OMNI samples were maintained.

### Metabolites stored in NS and OMNI samples at room temperature

**Figure 6:**
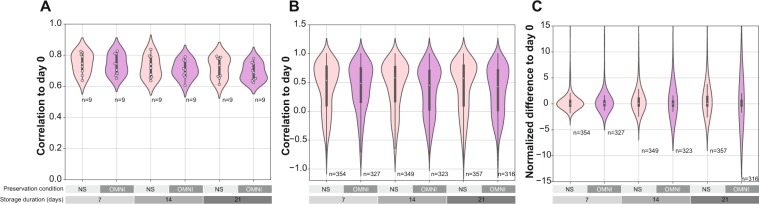
Changes in metabolite profiles during the periods of room temperature storage. (**A**) Correlations of the total metabolite profiles between immediately frozen samples and those at each time point. (**B**) Correlations of individual metabolite intensities between samples frozen immediately and at each time point. (**C**) Normalized differences of individual metabolite peaks between immediately frozen and each time point samples.

Next, we investigated changes in the metabolite profiles of the samples stored at room temperature. We first calculated Spearman’s correlations of the entire metabolite profiles between immediately frozen samples and those at each time point for each storage condition. The metabolite profiles of the samples stored at room temperature gradually deviated from those of the immediately frozen samples, but remained well correlated for up to 21 days in both NS and OMNI samples (Fig. 6A). To evaluate changes in the intensity relationship of a metabolite peak over time, we next examined the correlations of each metabolite peak between the immediately frozen samples and those at each time point. We found that a large portion of the metabolite peaks of the samples at each time point was highly correlated with the peaks of the immediately frozen samples in both NS and OMNI conditions (Fig. 6B). Furthermore, we investigated the changes in the absolute intensity of each metabolite peak by calculating the difference in the average peak intensity between samples at each time point and immediately frozen samples, and normalizing the value by dividing by its standard deviation. A time-dependent increase of deviation was observed in both OMNI and NS samples, but the degree of deviation was larger for the OMNI samples than for the NS samples (Fig. 6C). This storage time-dependent deviation was also observed in the principle component analysis of the metabolite profiles **(**Fig. S4**)**. The results indicate that absolute intensities of individual peaks change when the samples are stored at room temperature, but that the intensity relationships of peaks between the samples frozen immediately and those at each time point can be preserved over time regardless of the usage of preservation solution.

**Figure 7:**
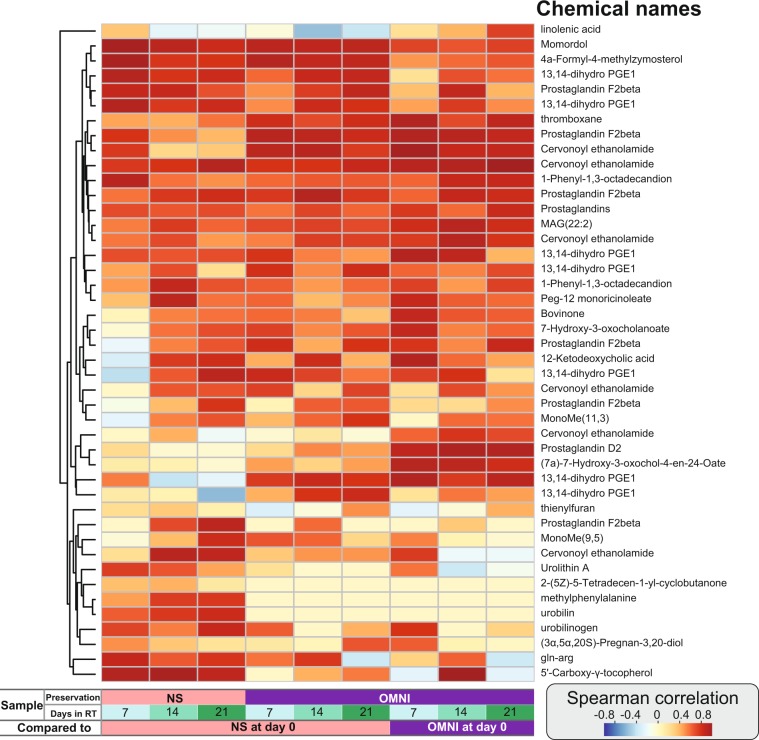
Signal decay of identified metabolites. Spearman correlations of identified metabolites between immediately frozen samples and those at each time point.

We further investigated which metabolites were not detected in the preservation solution and which metabolites can be stably detected over time. The metabolite corresponding to each peak was searched using the ChemSpider database^21^, and 44 metabolite peaks were identified as 27 unique metabolites. Among them, 2-(5Z)-5-tetradecen-1-yl-cyclobutanone, urobilin, and methylphenylalanine were observed only in the NS samples. The individual correlations between samples were examined for the identified metabolites **(**Fig. 7**)**. The abundances of metabolites such as momordol, 4a-formyl-4-methylzymosterol, and 13,14-dihydro-PGE1 showed high correlations between the immediately frozen samples and those at each time point in both NS and OMNI conditions, but the correlations were higher among NS samples **(**Fig. 7**)**. The Spearman correlations of metabolites such as thromboxane, prostaglandin F2beta, and cervonoyl ethanolamide between the samples that were immediately frozen and at each time point were higher in the OMNI condition than in the NS condition **(**Fig. 7**)**. In the identified metabolite peaks, the abundance relationships between the immediately frozen samples and those at each time point tended to be better conserved in the OMNI samples.

### Microbiome–metabolome associations in NS and OMNI samples

In the previous sections, we evaluated whether the preservation solution allows microbiome profiling and metabolite profiling, separately, and found that both microbiome and metabolite data can be obtained in the samples stored with the preservative solution at room temperature. In multi-omics studies, the two paired data can be used to infer microbe–metabolite associations; therefore, we further evaluated whether such associations can be reliably retrieved from the samples collected in the preservative solution. For each condition, we analyzed the microbe–metabolite associations by calculating Spearman’s correlation between the relative abundances of a microbe and the intensities of a metabolite peak. We then counted the numbers of significant associations (FDR <0.05) that were found in both immediately frozen samples and those at each time point. Only 2.7% of the associations were commonly observed in the immediately frozen NS and OMNI samples, and on average, 1.3% and 6.2% of associations were shared between the immediately frozen samples and those stored at room temperature in the NS and OMNI conditions, respectively **(**Table 1**)**. Because the number of samples used in this study was small, it is impossible to draw biologically meaningful and statistically sound conclusions from this association analysis, which is reflected in the small number of common associations reported here. Nevertheless, a four times higher overlap of the associations was found in the OMNI samples than in the NS samples, suggesting the possibility of finding microbe–metabolite associations in fecal samples collected in the preservative solution.

**Table 1:** Co-occurrence of metabolite-microbiota associations.

Comparison target			Co-occurrence (Jaccard index, %)	
Preservation condition	Storage duration (days)	N	vs. NS 0 (N = 246)	vs. OMNI 0 (N = 167)
NS	0	246	246 (100)	11 (2.7)
NS	7	50	1 (0.34)	2 (0.93)
NS	14	65	3 (0.97)	0 (0.0)
NS	21	111	9 (2.6)	3 (1.1)
OMNI	0	167	11 (2.7)	167 (100)
OMNI	7	53	0 (0.0)	11 (5.3)
OMNI	14	92	8 (2.4)	11 (4.4)
OMNI	21	202	9 (2.1)	30 (8.8)

Metabolite-microbiota associations were measured by Spearman’s correlation for each preservation conditions. A false discovery rate (FDR) of <0.05 was considered to be statistically significant. The numbers of the significant associations in each condition are shown. The numbers of the significant associations shared by each condition and immediate frozen NS or OMNI and their Jaccard indexes are also shown.

## Discussion

This study shows that microbiome compositions are stably maintained for up to three weeks when fecal samples are collected in OMNIgene-GUT solution at room temperature. We found that, with those samples, metabolites in fecal materials can also be measured and relative abundance relationships of part of them can be preserved during the period of storage at room temperature, suggesting that it is possible to extract both microbiome and metabolite data from a single sample. The simultaneous acquisition of both microbiome and metabolite information allows researchers to reveal the associations between microbes and metabolites, and can lead to a deeper understanding of how gut microbes modulate host health. However, our research also reveals the potential weakness of collecting samples for metabolite studies using the preservation solution. First, the organic solvent in the preservation solution can affect the elution of specific chemicals. Second, the solution may lead to intensity changes of some metabolites. Therefore, when a study targets specific metabolites in fecal samples, it should be checked whether they can be detected after the fecal sample is mixed with a preservation solution.

It should be noted that this pilot study was performed with the fecal samples from a limited number of individuals. Therefore, we cannot claim that the microbiome and metabolome profiles observed here represent those from other larger numbers of individuals. In the fecal samples from other individuals, the degree of changes in the microbiome and metabolome profiles due to the sample storage can be larger or smaller than those reported in this study. Therefore, future studies with a larger number of samples from various populations should be conducted to confirm the results in our pilot study.

In conclusion, our study showed that a fecal sample collected in a single collection procedure and stored at room temperature can be used for both microbiome and metabolite analyses. Although this approach has several limitations for metabolite analysis, we anticipate that a number of meaningful microbe–metabolite associations can be found from fecal samples collected in the preservative solution in population-level studies, which would lead to an explanation of how gut microbes affect host health via metabolites.

## Methods

### Fecal sample collection

Fecal samples were obtained from three participants of the Korean gut microbiome project. These participants were in their mid-twenties and their health status was normal. This study was approved by the institutional review board of Theragen ETEX Bio Institute (700062-20160804-JR-005-02). All experiments and analyses were executed in accordance with the approved guidelines and relevant regulations, and informed consent was obtained from each participant. Fecal samples of approximately 20 g were collected from each participant in a sterile stool collection container. After homogenizing each fecal sample with a sterile spatula, fecal samples were immediately aliquoted into 24 cryotubes each containing 0.5 g (non-stabilized [NS] samples). The remaining fecal material was mixed with OMNIgene-GUT stabilizing solution by transferring the fecal sample into the yellow tube top of four OMNIgene-GUT tubes (OMR-200; DNA Genotek, Ontario, Canada) and shaking for a minimum of 30 s. The mixtures of fecal material and OMNIgene-GUT solution were pooled into a Falcon tube, and then aliquoted into 24 cryotubes each containing 0.3 g (OMNIgene-GUT solution-treated [OMNI] samples). Aliquots were frozen at −80 °C on the day of collection and after 7, 14, and 21 days of room temperature storage. A total of six aliquots were prepared for each storage condition (with and without stabilizing solution and different lengths of time at room temperature). Three aliquots each were used for microbiome and metabolome analyses.

### Microbiome analysis

#### 16S rRNA gene sequencing

DNA extraction was performed using the QIAamp DNA Stool Mini Kit (Qiagen, Hilden, Germany) with the following modifications^22^. Briefly, 250 µL of the fecal sample was placed into a 2-mL tube containing 0.3 g sterile 0.1 mm zirconia beads (BioSpec, OK, USA) with 1.2 mL ASL lysis buffer from QIAamp DNA Stool Mini Kit (Qiagen, Hilden, Germany) and vortexed for 2 min. The samples were subsequently heated at 95 °C for 15 min and then subjected to two cycles of bead beating at a frequency of 30 Hz for 1 min using the Qiagen TissueLyser II. After centrifugation, 1.2 mL of the supernatant was treated with an InhibitEX Tablet, and 350 µL of the resulting supernatant was used in the subsequent steps with a QIAcube system (Qiagen, Hilden, Germany). Total DNA was eluted in 200 μL of elution buffer and stored at −20 °C until use.

Library preparation of the V3–V4 hypervariable region of the 16S rRNA gene was performed according to the 16S Metagenomic Sequencing Library Preparation Illumina protocol (Part #15044223 Rev. B; Illumina, San Diego, CA, USA). The library pool containing equal molar quantities of each sample was sequenced using the MiSeq. 2 × 300 system (Illumina).

#### Data processing

The presence of the primer sequences was checked in the raw sequence reads, and read pairs without either forward or reverse sequences were removed. Then, amplicon sequence variant (ASV) tables were generated using the DADA2 method^23^ in QIIME2 (2019.01 version)^24^. Forward and reverse truncation lengths were set to 270 and 220, respectively. The taxonomy of each ASV was determined using the Naïve Bayesian classifier^25^ in QIIME2, trained on the V3-V4 region using the Greengenes 13.8 database^26^.

Diversity and principle coordinates (PCoA) analyses were performed with the rarefied ASV table containing 10,000 sequences per sample in QIIME2 (2019.01 version)^24^. The alpha diversity (observed species, Pielou evenness, and Shannon index) and beta diversity (Bray–Curtis distance) metrics were calculated, and PCoA was performed with Bray–Curtis distance.

### Metabolite analysis

#### Analysis of metabolite profiles by liquid chromatography (LC)–mass spectrometry (MS)

Lyophilized fecal samples were mixed with 50% methanol solution containing the internal standard terfenadine and homogenized using a bullet blender (Next Advance, Troy, NY, USA). After centrifugation, the supernatant was analyzed by ultra-performance (UP)LC quadrupole time-of-flight (Q-TOF) MS on an Acquity UPLC-Q-TOF instrument (Waters, Milford, MA, USA) equipped with an Acquity UPLC BEH C18 column (2.1 mm × 100 mm, 1.7 μm; Waters), with the column temperature set at 40 °C. A mixture of deionized water containing 0.1% formic acid and acetonitrile containing 0.1% formic acid was used as the mobile phase, and flow rate was set to 0.35 ml/min. LC column eluents were detected by Q-TOF MS in positive electrospray ionization mode. The desolvation gas flow rate was set to 800 L/h at a temperature of 400 °C and the source temperature was set to 100 °C. Capillary and sampling cone voltages were set to 3 kV and 30 V, respectively. TOF MS data were obtained in the scan range between *m*/*z* 100 and 1500 with a scan time of 0.2 s. Leucine-enkephalin ([M + H] = 556.2771) was used as the lock mass at a frequency of 10 s. For quality control, a mixture of all samples was analyzed once every 20 samples. Tandem MS data were obtained in the *m*/*z* 50–1500 range with a collision energy ramp from 10 to 30 eV.

#### Data processing

MarkerLynx software was used for collection, normalization, and alignment of UPLC-Q-TOF MS data. Peaks were identified using a peak-to-peak baseline noise of 1, noise elimination of 6, peak-width at 5% height of 1 s, and an intensity threshold of 10,000. Metabolite data were evenly arranged with a mass window of 0.05 Da and a retention time window of 0.2 min. All data were normalized to the internal standard. Metabolites were identified using the Chemspider database of Unifi v.1.8.2.169 software (Waters).

### Bioinformatics analysis

#### Analysis of microbiome data

The significance of differences in alpha and beta diversity between NS and OMNI samples was evaluated with a *t*-test at each time point. A linear mixed model analysis was carried out to identify taxa that were significantly altered by storage time in each storage condition^4^. The changes in microbial composition were also analyzed by calculating log_2_-fold changes in the relative abundance to the immediately frozen samples at each time point.

#### Analysis of metabolite peaks

A metabolite peak was assumed to be present in a sample if its measured intensity was larger than 0. A set of peaks was obtained for each sample, and the size of intersection sets between two samples was calculated to identify shared metabolites. The similarity between detected peaks was evaluated as Jaccard similarity (i.e., the number of elements in the intersection set divided by the number of elements in the union set).

#### Relative correlations of individual metabolite peaks

The intensity of an individual peak in the two different groups was determined and the Spearman correlation coefficient was calculated. To evaluate differences in peak intensity between the two preservation conditions, we calculated the correlation between the peak intensity values in the day 0 NS and OMNI samples. To evaluate changes in metabolite levels during the storage at room temperature, we compared metabolite peak intensities of day 7, 14, and 21 samples to those of day 0 samples.

#### Metabolite peak intensity deviation analysis

The shift in absolute intensity of individual peaks was calculated as the average difference between peaks in day 0 samples and those in day 7, 14, and 21 samples. For a collective comparison of individual peaks, the peak intensity difference was normalized by dividing the value by the standard deviation of day 0 data.

#### Principle component analysis

The metabolite profiles were normalized by dividing the peak intensities by their summation. Principle components that segregated the samples were determined using the PCA module in the Scikit-learn package (Version 0.20.3)^27^. Metabolite peak intensities between NS and OMNI samples largely differed in the total magnitude. Herein, because the coordinates in PCA were primarily affected by the total magnitude of each sample, we considered the relative abundance of metabolites to focus on differences in metabolite profiles among samples, regardless of the total magnitude.

#### Analysis of metabolite–microbe association

For each condition, the associations of metabolite–microbe pairs were statistically evaluated by calculating Spearman’s correlation coefficients. A false discovery rate (FDR) of <0.05 was considered to be statistically significant. The numbers of significant metabolite–microbe associations that were commonly found in each condition and in the immediate frozen NS or OMNI were counted and their Jaccard indexes (%) were calculated.

#### Software

For data processing and statistical analysis, Python (Version 2.7.15) and R (Version 3.6.0) were utilized. Pandas (Version 0.23.1)^28^ was used for data processing, SciPy (Version 0.17.1)^29^ was used for the calculation of Spearman’s correlation, and Matplotlib (Version 2.2.3)^30^, R, and GraphPad Prism were used to visualize the data.

## Supplementary information

Supplementary Information.

## Data Availability

All raw sequencing data described in this study are available from the European Nucleotide Archive (ENA) under accession number PRJEB31155. The metabolite profile is available from Zenodo (10.5281/zenodo.3462877).
