# Supplementary material for: Changes in microbiome and metabolomic profiles of fecal samples stored with stabilizing solution at room temperature: a pilot study
Source: Sci Rep. 2020 Feb 4;10:1789. doi: 10.1038/s41598-020-58719-8 (PMC7000387; doi:10.1038/s41598-020-58719-8)
Supplement: Supplementary file 1 — Supplementary Information. [file 41598_2020_58719_MOESM1_ESM.docx]

**Supplementary Information**

**Changes in microbiome and metabolomic profiles of fecal samples stored with stabilizing solution at room temperature: a pilot study**

Mi Young Lim^1,†^, Seungpyo Hong^1,†^, Bo-Min Kim^2^, Yongju Ahn^3^, Hyun-Jin Kim^2,4^, Young-Do Nam^1,5,*^

^1^Research Group of Healthcare, Korea Food Research Institute, Jeollabuk-do 55365, Republic of Korea

^2^EZmass Co., Ltd., Gyeongsangnam-do 52828, Republic of Korea

^3^Theragen Etex Bio Institute, Gyeonggi-do 16229, Republic of Korea

^4^Department of Food Science and Technology, Division of Applied Life Sciences (BK21 Plus), Institute of Agriculture and Life Science, Gyeongsang National University, Gyeongsangnam-do 52828, Republic of Korea

^5^Department of Food Biotechnology, Korea University of Science and Technology, Daejeon 34113, Republic of Korea

^†^These authors contributed equally to this work.

^*^Corresponding author. Email: [youngdo98@kfri.re.kr](mailto:youngdo98@kfri.re.kr)


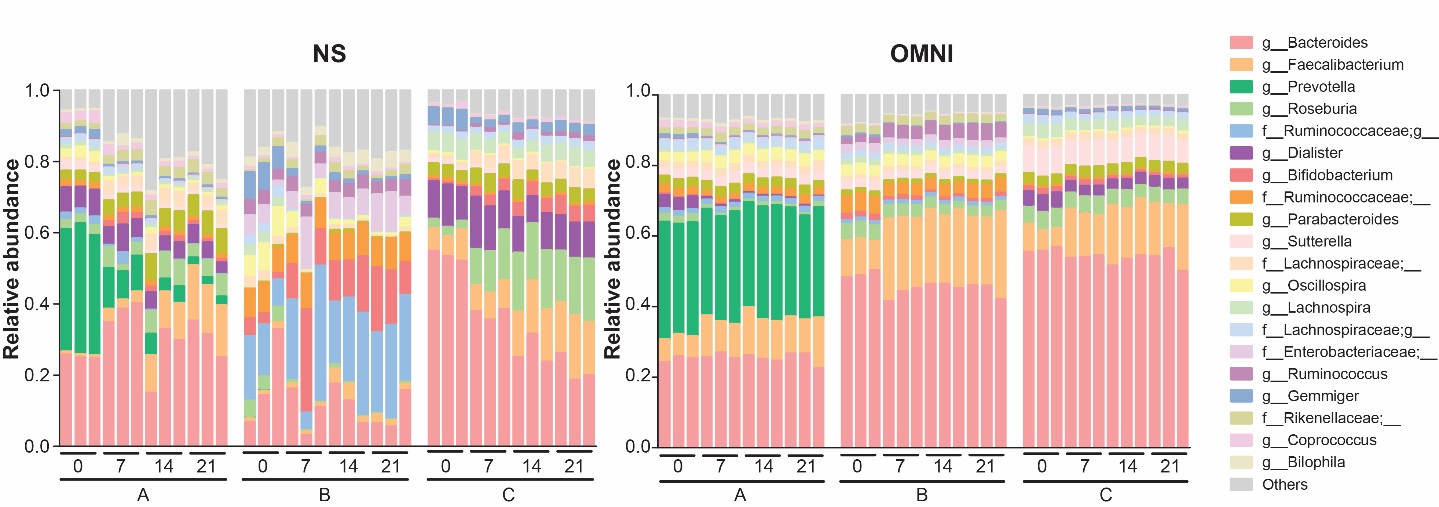


**Supplementary Figure S1. Relative abundance of the top 20 most abundant genera.**


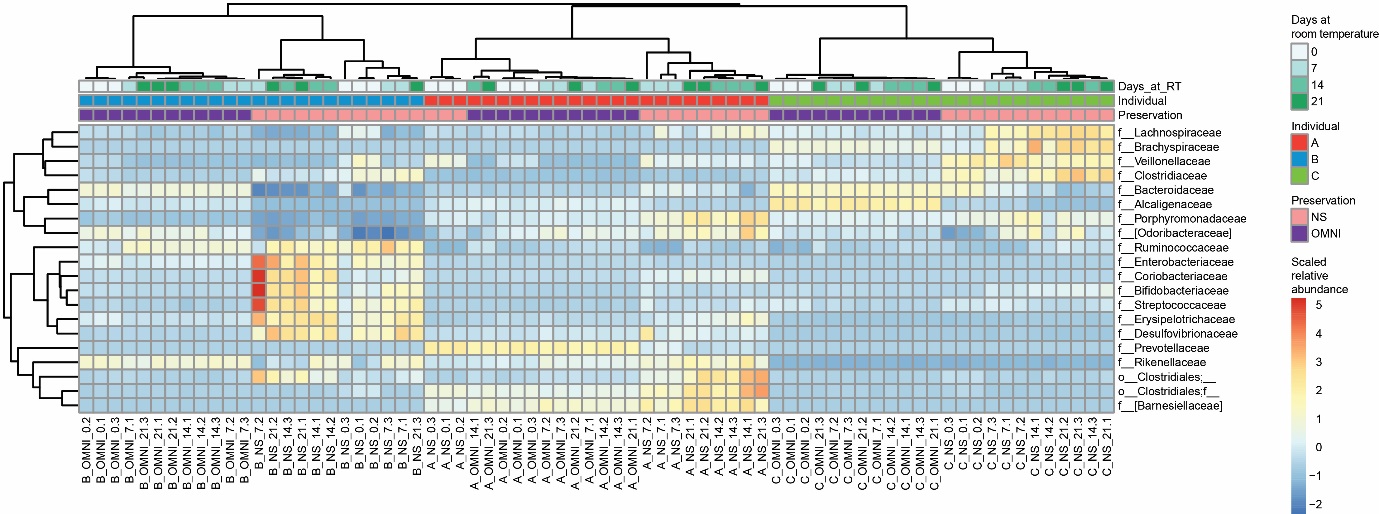


**Supplementary Figure S2. Clustering with microbiota composition.** Samples were clustered with their microbiota composition determined at family level, or at order level for cases where family could not be assigned. The relative abundances of taxa were scaled for clear visualization, specifically by subtracting the sample mean value and dividing by the sample standard deviation. The data were clustered with a hierarchical clustering method using Pearson correlation as the distance measure and using the complete-linkage criterion.


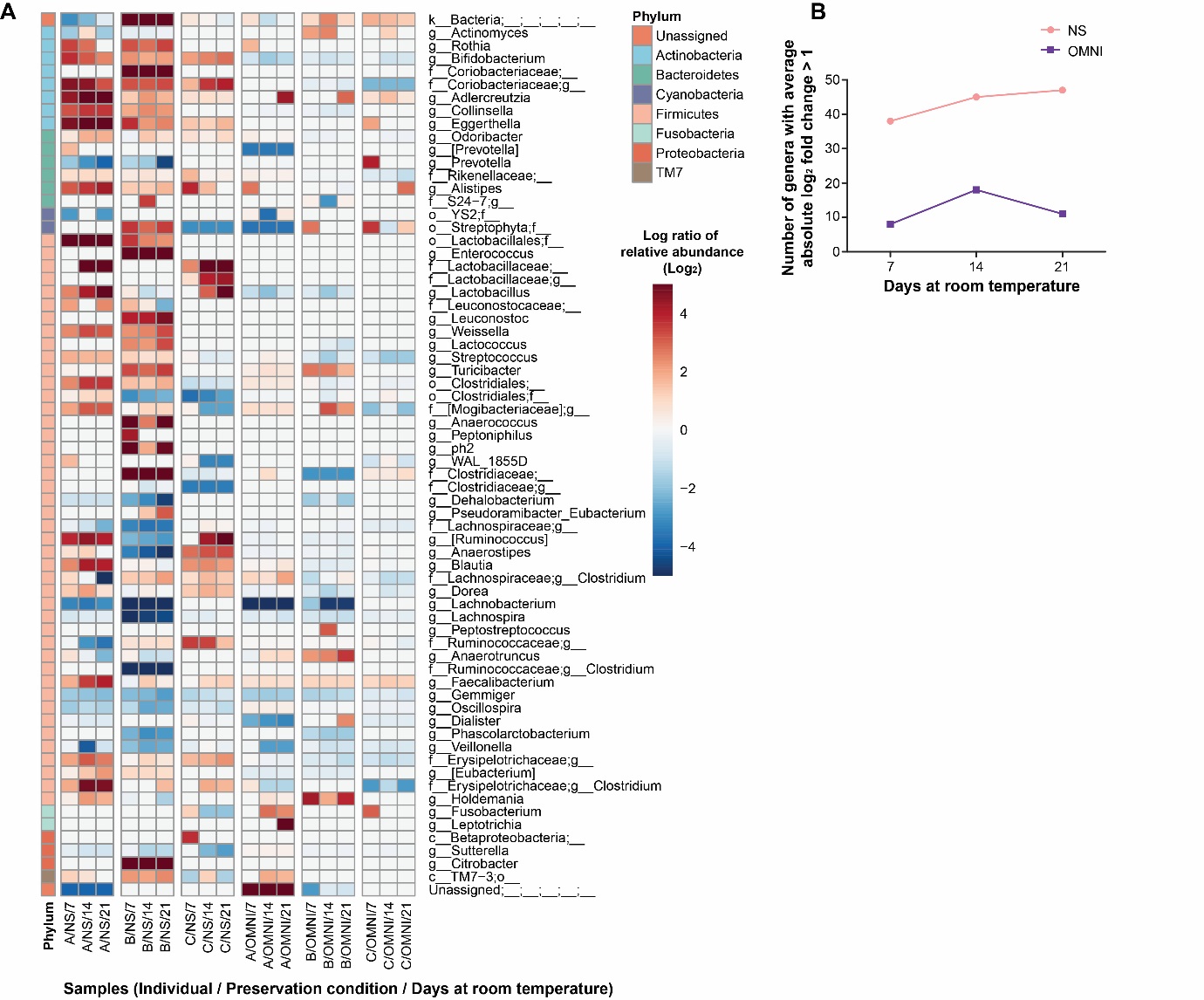


**Supplementary Figure S3. Log_2_-fold changes in the microbial relative abundance of the samples at each time point to the immediately frozen samples**. (A) The genera with average absolute log_2_-fold changes >1 for at least one preservation condition are displayed. (B) For each preservation condition, the numbers of genera with average absolute log_2_-fold changes >1 are plotted at each time point.


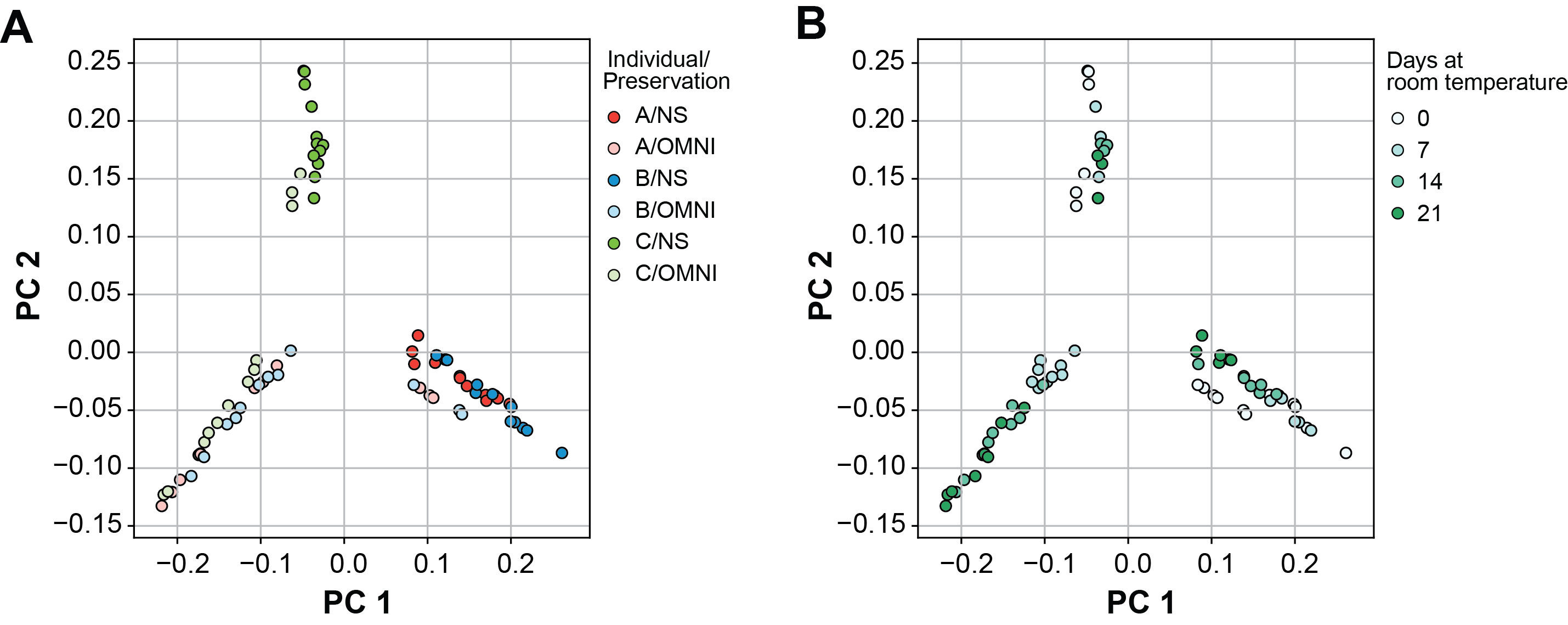


**Supplementary Figure S4. Principle component analysis of the metabolite profiles.** Samples are colored by individual and storage condition (A) and by days stored at room temperature (B).
